# THE APPLICATION OF TIME BANKING IN ELDERLY CARING: A MIXED-STUDIES SYSTEMATIC REVIEW

**DOI:** 10.1093/geroni/igac059.2358

**Published:** 2022-12-20

**Authors:** Hui Ding, Hui-Ling Li

**Affiliations:** Soochow University, Suzhou, Jiangsu, China (People's Republic); School of Nursing, Suzhou Medical College, Soochow University, Suzhou, Jiangsu, China (People's Republic)

## Abstract

Aging has become a universal concern of the world, and its process is accelerating, which leads to the shortage of the care resources and increased burden of social development. Based on this problem, The concept of time banking has been gradually applied to geriatric caring, forming a model of mutual support for older people, which has been practiced in different countries. However, there is no consensus about the effect of time banking for older person. Thus, the aim of this review was to integrate research on the application of time banking to summarize the benefits for older people and challenges faced in different countries. Moreover, the review put forward solutions to provide references for the localization of time banking in China. PubMed, Wiley Online Library, Web of Science, SAGE Journals Online, Science Direct, EbscoHost, SpringerLink and CNKI databases were searched to include 22 eligible studies. Qualities of included studies were assessed, evidences about the benefits for older person and challenges regarding time banking was synthesized. Although the overall study quality was medium, consistent evidence was existed to indicate that time banking has beneficial effects which containing social values and personal values on the older people. Furthermore, the comprehensive evidences also showed that time banking would be improved in the following four aspects: diversity of volunteers, improvement of service quality, variety of incentives and scientific management. Additional well-designed and high-quality studies are needed for us to better understand the opportunities and challenges of time banking.

